# γ-Secretase in Alzheimer’s disease

**DOI:** 10.1038/s12276-022-00754-8

**Published:** 2022-04-08

**Authors:** Ji-Yeun Hur

**Affiliations:** Chemical Biology Program, Memorial Sloan Kettering Cancer Center, New York, NY 10065 USA

**Keywords:** Alzheimer's disease, Alzheimer's disease

## Abstract

Alzheimer’s disease (AD) is caused by synaptic and neuronal loss in the brain. One of the characteristic hallmarks of AD is senile plaques containing amyloid β-peptide (Aβ). Aβ is produced from amyloid precursor protein (APP) by sequential proteolytic cleavages by β-secretase and γ-secretase, and the polymerization of Aβ into amyloid plaques is thought to be a key pathogenic event in AD. Since γ-secretase mediates the final cleavage that liberates Aβ, γ-secretase has been widely studied as a potential drug target for the treatment of AD. γ-Secretase is a transmembrane protein complex containing presenilin, nicastrin, Aph-1, and Pen-2, which are sufficient for γ-secretase activity. γ-Secretase cleaves >140 substrates, including APP and Notch. Previously, γ-secretase inhibitors (GSIs) were shown to cause side effects in clinical trials due to the inhibition of Notch signaling. Therefore, more specific regulation or modulation of γ-secretase is needed. In recent years, γ-secretase modulators (GSMs) have been developed. To modulate γ-secretase and to understand its complex biology, finding the binding sites of GSIs and GSMs on γ-secretase as well as identifying transiently binding γ-secretase modulatory proteins have been of great interest. In this review, decades of findings on γ-secretase in AD are discussed.

## Alzheimer’s disease: changing activity of critical enzyme offers therapeutic option

Drugs that only affect the activity of an enzyme called γ-secretase but do not entirely block its function could provide a safer therapeutic option for preventing the build-up of toxic proteins in the brain linked to AD. Dr. Ji-Yeun Hur from Memorial Sloan Kettering Cancer Center, New York, USA, discusses the functions of γ-secretase, which include cleaving amyloid precursor proteins into smaller fragments that can aggregate to form sticky plaques, and reviews attempts to develop therapies directed against the enzyme. Early drug candidates proved too toxic because they blocked all functions of γ-secretase including desirable functions, leading to side effects. Newer drug strategies have focused on changing how γ-secretase processes Alzheimer’s-related proteins without disrupting its other physiological functions. A better understanding of γ-secretase’s complex structure and various functions could inform those strategies.

## Introduction

Alzheimer’s disease (AD) is the most common form of dementia^1^. Two major pathological hallmarks of AD are senile plaques, which result from extracellular accumulation and deposition of amyloid β-peptide (Aβ), and neurofibrillary tangles containing the hyperphosphorylated tau protein in neurons^2,3^. AD progresses slowly, and the progression is estimated to occur 25 years prior to the onset of symptoms^4^. The current treatment for AD is to use acetylcholinesterase inhibitors and the *N*-methyl-d-aspartate receptor antagonist memantine for the symptomatic improvement of AD^5^_,_ and there is no cure available. Recently, aducanumab targeting Aβ aggregates in the brain^6^ was approved with some controversy.

According to the amyloid cascade hypothesis, the accumulation of Aβ in the brain is the primary cause of AD^7^. The chronic imbalance between the production and clearance rate of Aβ may lead to increased Aβ42 levels, followed by Aβ oligomerization, fibril formation, and accumulation in plaques^7^. Both Aβ oligomers and plaques damage neurons by astrocytic activation, oxidative injury, and altered kinase/phosphatase activities, followed by the formation of neurofibrillary tangles^7^. Therefore, therapeutics aimed at lowering Aβ levels could be clinically useful for the treatment of AD^7^. Genetically inherited familial Alzheimer’s disease (FAD) genes also support Aβ as the key driver in the amyloid cascade hypothesis. In most cases, *APP* mutations increase the ratio of Aβ42/Aβ40 or total Aβ production^8^. Missense mutations, insertions, or deletions in *PSEN* are mostly located in the transmembrane regions or hydrophilic loops in the cytosol, and they result in an increased ratio of Aβ42/Aβ40^8^.

## APP processing and Aβ

**Fig. 1:**
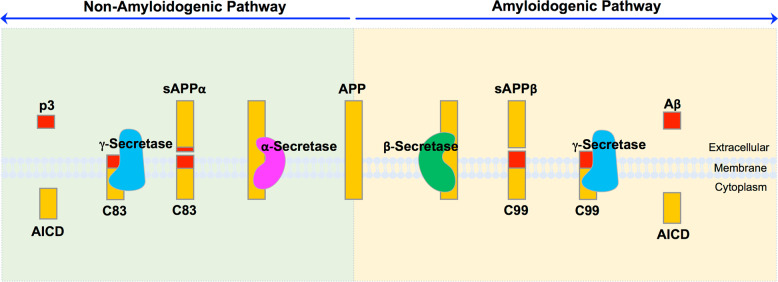
APP processing. In the amyloidogenic pathway, β-secretase cleaves APP extracellularly to release sAPPβ and a membrane-bound APP-CTF (C99). C99 is subsequently cleaved by γ-secretase to release Aβ and the APP intracellular domain (AICD). In the non-amyloidogenic pathway, APP is cleaved by α-secretase to release sAPPα and a membrane-bound APP-CTF (C83). C83 is cleaved further by γ-secretase to release p3 and AICD.

The amyloid plaques in the brains of AD patients consist of fibrils formed by Aβ. Aβ is produced from amyloid precursor protein (APP) by sequential proteolytic cleavages of β-secretase (β-site APP-cleaving enzyme, BACE) and γ-secretase (Fig. 1)^9^. In the amyloidogenic pathway of APP processing, APP is initially cleaved by BACE, resulting in soluble APPβ (sAPPβ) and membrane-bound APP-CTF (C99)^9^. C99 is further cleaved by γ-secretase to release Aβ extracellularly and the APP intracellular domain (AICD) for nuclear translocation^9^. Alternatively, APP is cleaved by α-secretase to produce sAPPα and APP-CTF (C83) (Fig. 1)^9^. C83 is further cleaved by γ-secretase to produce p3 and AICD^9^.

**Fig. 2:**
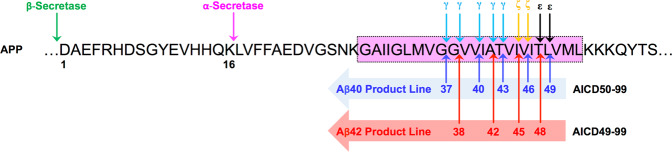
γ-, ζ-, and ε‑Cleavage sites for Aβ species. After APP is cleaved by β-secretase, APP-CTFs are processed by ε-cleavage, resulting in Aβ49 and AICD50-99 or Aβ48 and AICD49-99. Aβ49 is further cleaved at the ζ-site to Aβ46, and the Aβ40 product line follows (Aβ49→46→43→40→37). The Aβ42 product line is Aβ48→45→42→38. The β-, α-, γ-, ζ-, and ε-cleavage sites are indicated by arrows. Membranes are indicated in pink. Aβ sequence numbering starts from 1 (after β-secretase cleavage) to 49 (after ε-cleavage).

It has been commonly referred to as the γ-secretase cleavage to release Aβ40 or Aβ42, and AICD. The γ-secretase cleavage site can be further separated into γ-, ζ-, and ε-cleavage sites (Fig. 2)^10^. The γ-site ends at Aβ40 or Aβ42, and AICD starts at Aβ49 or Aβ50. This discrepancy with missing amino acid residues led to the new identification of the ε-cleavage site at Aβ49^11–14^. The question of whether the γ- and ε-cleavages occur sequentially or independently from each other was answered by a new identification of the ζ-cleavage site at Aβ46^15,16^. Aβ peptides are cleaved mainly by tripeptide trimming via the Aβ40 product line (Aβ49→46→43→40→37) or the Aβ42 product line with the last cleavage step by tetrapeptide trimming (Aβ48→45→42→38) (Fig. 2)^17^. In addition, other Aβ peptides found in varying lengths support the link between two major Aβ40 and Aβ42 product lines and multiple interactive pathways releasing tri-, tetra-, penta-, and hexapeptides^18,19^.

The physiological role of Aβ is not yet clear. The length of Aβ found in CSF or brain varies from 37 to 43 amino acids^20–22^. Aβ42 is more prone to aggregate and more toxic than Aβ40, even though the ratio of production for Aβ42 and Aβ40 is approximately one to nine^23^. Aβ42 is the major component of amyloid plaques^23–25^, and Aβ43 was reported to exist in amyloid deposition of the human AD brain^20^.

### γ-Secretase

**Fig. 3:**
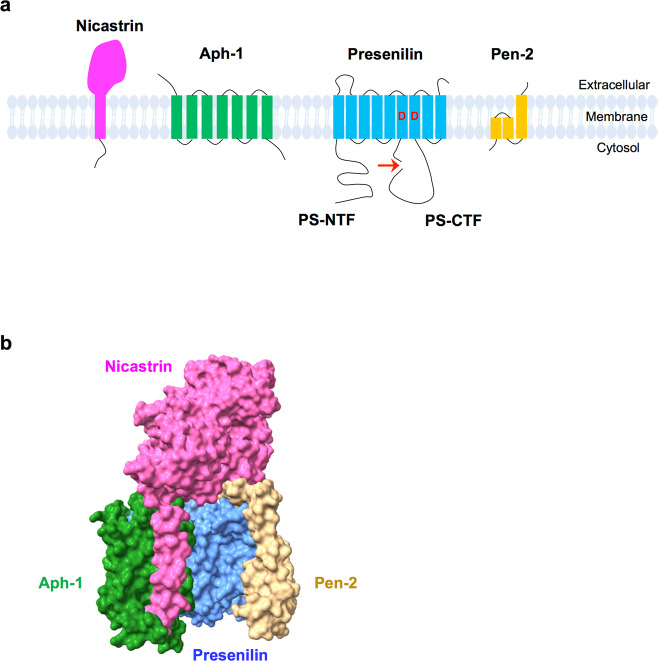
The γ-secretase complex. **a** γ-Secretase complexes require at least four essential components: presenilin (PS), nicastrin (Nct), Aph-1, and Pen-2. The two catalytic aspartyl residues in PS are indicated by ‘D’ (Asp257 in TM6 and Asp385 in TM7). PS undergoes endoproteolysis (indicated by arrow) and becomes a PS-NTF/PS-CTF heterodimer. **b** The γ-secretase complex structure is shown in the surface view. Presenilin (blue), nicastrin (magenta), Aph-1 (green), and Pen-2 (yellow). Rendered from Protein Data Bank entry 7D8X. The structural figure was prepared with UCSF ChimeraX 1.2.5.

γ-Secretase carries out a sequential cleavage of the substrate C99 to generate Aβ peptides^9^. As such, γ-secretase has been an attractive target for the potential treatment of AD. However, it has been found to be challenging, and more studies are needed to fully understand γ-secretase. γ-Secretase is a transmembrane protein complex containing presenilin (PS), nicastrin, anterior pharynx defective-1 (Aph-1), and presenilin enhancer-2 (Pen-2) (Fig. 3a, b). γ-Secretase belongs to a new class of proteases, intramembrane-cleaving proteases (I-CliPs), and its unusual cleavage processes substrates in the lipid bilayer of membranes^26^.

### PS, nicastrin, Aph-1, and Pen-2

Compared to BACE, γ-secretase is not strictly site-specific and yields Aβ peptides that are 37–43 amino acids long^20–22^. The topology of PS has nine transmembrane spanning domains^27^. PS has two mammalian homologs, PS1 and PS2, which share 67% sequence similarity^28^. γ-Secretase is an aspartyl protease with essential aspartyl residues at positions 257 and 385 within transmembrane domains 6 and 7 of PS (both PS1 and PS2) that constitute the active site of the protease (Fig. 3a)^29,30^. The mature form of PS is cleaved endoproteolytically between the sixth and the seventh transmembrane domains into an N-terminal and a C-terminal fragment (NTF and CTF), and the PS1-NTF/PS1-CTF heterodimer forms the catalytic site of γ-secretase^30–33^. Transition state analog (TSA) γ-secretase inhibitors (GSIs) that bind to PS1-NTF and PS1-CTF also support this finding^34,35^. In addition, >300 *PSEN*-harboring FAD mutations increased the Aβ42/40 ratio, and knockout (KO) of *PSEN1* decreased γ-secretase cleavage of APP and reduced Aβ production^31^.

Nicastrin was discovered by its association with PS after immunoaffinity purification using an anti-PS antibody^36^. Two additional cofactors, Aph-1 and Pen-2, were discovered by genetic screening in *Caenorhabditis elegans*^37,38^. Nicastrin is a single-pass transmembrane protein with a large extracellular domain. Immature nicastrin is ~110 kDa, and the apparent molecular weight is increased to ~130 kDa after *N*-glycosylation in the Golgi/TGN compartments^39^. This mature form of nicastrin is associated with the active γ-secretase complex^39–42^. Aph-1 is required for the cell-surface localization of nicastrin^38^, and Pen-2 is required for both the expression of PS and the maturation of nicastrin^43^.

The mRNA and protein expression of the γ-secretase complex subunits is ubiquitously expressed in the body^44^. The physiological functions of γ-secretase complex subunits were studied by using KO mice. PS1 KO mice are lethal, resulting Notch signaling deficiency, while the phenotype of PS2 KO mice is normal, and double KO of PS1 and PS2 is embryonic lethal, showing a severe Notch deficiency^45,46^. Nicastrin KO mice showed a Notch phenotype with embryonic lethality^47^. Aph-1a KO mice showed embryonic lethality, and Aph-1b/c KO mice (equivalent to human Aph-1b loss) showed reduced APP processing in several regions in the adult brain^48^. A KO study in zebrafish showed that Pen-2 is important for neuronal cell survival and protects cells from apoptosis^49^.

Whether these four subunits of γ-secretase are essential for its activity was investigated. The γ-secretase activity was reconstituted in *Saccharomyces cerevisiae*, which lacks endogenous γ-secretase activity, by co-expressing PS, nicastrin, Aph-1, and Pen-2^50^. Thus, these four proteins appear to be necessary and sufficient for γ-secretase activity^50^. This was also shown in *Drosophila* and mammalian cells^50–54^. Co-expression of all four components also increased the PS heterodimeric form, fully glycosylated nicastrin, and γ-secretase activity in mammalian cells^52^. In a postmortem human brain study, it was shown that human brain-derived γ-secretase is present as a high molecular weight protein complex containing PS, nicastrin, Aph-1, and Pen-2 and that these are associated with γ-secretase activity^55^. The activity of the γ-secretase complex was inhibited by the specific GSI L-685,458, suggesting that this γ-secretase complex isolated from the human brain is functional^55^.

The assembly of the γ-secretase complex is initiated in the endoplasmatic reticulum (ER)^56^_,_ where Aph-1 and nicastrin interact, followed by the binding of PS^53^. Thereafter, Pen-2 binds to the complex and facilitates the endoproteolysis of PS to PS-NTF and PS-CTF, resulting in an active γ-secretase complex^53^. In a γ-secretase activity study using a biotinylated affinity ligand, it was confirmed that PS heterodimers and mature nicastrin exist in the active enzyme complex^57^. It was also reported that bacterially synthesized recombinant proteins in liposomes such as PS1-ΔE9 (FAD mutation with PS1 exon 9 deletion) alone or PS1-full-length (FL)/Pen-2 have active γ-secretase activity^58^.

### Stoichiometry of γ-secretase

PS has two homologs, PS1 and PS2. Aph-1 has two homologs, Aph-1a and Aph-1b, in humans and one additional homolog, Aph-1c, in rodents. Aph-1a has two alternatively spliced forms, Aph-1aL (long form) and Aph-1aS (short form). In total, γ-secretase can form six different complexes in humans^44^.

The molecular weight of the four components is PS1-NTF (~30 kDa), PS1-CTF (~20 kDa), fully glycosylated nicastrin (~130 kDa), Aph-1 (~30 kDa), and Pen-2 (~12 kDa). The molecular weight of the γ-secretase complex is calculated to be ~220 kDa at a stoichiometry of 1:1:1:1 (PS:glycosylated nicastrin:Aph-1:Pen-2). Different methods have been used for the preparation and analysis of the complex, resulting in observed molecular weights in the range of 200–2000 kDa^50,52,55,59,60^. The lowest reported molecular weight of the complex is 200–250 kDa, corresponding to a monomeric complex^52^. The complex at ~440 kDa suggests a possible stoichiometry of 2:2:2:2^50^. Super-resolution imaging showed that a stoichiometry of 1:1 (PS1:NCT) at the cell surface and a BN-PAGE gel showed γ-secretase complexes at ~440 kDa^60^. In membranes from the postmortem human brain, the γ-secretase components were eluted in a fraction of > 1000 kDa^55^. Sato et al.^61^ reported the stoichiometry of active γ-secretase complexes as 1:1:1:1. Differences in the molecular weight of the γ-secretase complex might indicate the possibilities of additional proteins, either novel core components or proteins binding transiently (γ-secretase modulatory protein, GSMP). The molecular weight of the γ-secretase complex with TMP21 was reported to be approximately 660 kDa^62^. Another binding protein, GSAP co-eluted with γ-secretase complex components at ~670 kDa^63^. Active γ-secretase complexes captured by Compound 3 showed a GSMP, Hif-1α, with γ-secretase complexes in high molecular weight fractions^64^.

### Trafficking and localization of γ-secretase

How APP, BACE1, and γ-secretase are trafficked and processed through subcellular compartments has been studied to identify the sites for Aβ production in cells. Aβ is found in the TGN^65^ and endosomes^66^. The subcellular localization of Aβ in brain tissue is mainly endosomal as well^67,68^. APP is cleaved by α-secretase at the cell surface^69^, while BACE1 cleavage occurs mostly in the late Golgi/TGN and endosomes^70^. γ-Secretase components have been found in many subcellular compartments, such as the ER, ER-Golgi intermediate compartment, Golgi, TGN, endosomes, and plasma membrane^71–74^. Interestingly, PS was also found in synaptic compartments^75–78^. In addition, all four γ-secretase components were found in phagosomes^79^. PS1, nicastrin, and APP are localized in the outer membranes of lysosomes^80^.

Importantly, the sites for γ-secretase activity have been investigated. A biotinylated active site probe labeled γ-secretase in the plasma membrane of cells^73,81^. Additionally, a small fraction of active γ-secretase was found in mitochondria^82^. γ-Secretase enriched in endosomes, in the plasma membranes, and at synapses is active to produce Aβ or AICD, and active γ-secretase was labeled by a GSI in the brain or primary cortical neurons^74,83^.

The lipid membrane environment can also affect the activity of proteins. Since γ-secretase is a transmembrane-bound protein, different detergents have been used to extract proteins from membranes and study the complex. However, γ-secretase can also be studied in a membrane environment, preserving some of its natural interactions with lipids. Cholesterol and sphingolipids are the major lipid constituents of ordered microdomains called lipid rafts in cell membranes^84^. Lipid rafts are considered to be dynamic platforms for cell signaling, membrane protein sorting, and transport^84^. Several findings suggest that the trafficking and processing of APP are regulated in lipid rafts^85–89^. APP, BACE, and γ-secretase have been shown to localize to lipid rafts. APP and BACE residing in separate lipid rafts can merge in endosomes, where amyloidogenic processing occurs^85^. Active γ-secretase was found in lipid rafts^86^ and brain lipid rafts^90^, and γ-secretase was active in lipid rafts from post-Golgi compartments and endosomes^87^. The reconstitution study of γ-secretase with different lipid mixtures showed that a lipid raft-like condition gave the highest γ-secretase activity^91^.

A recent high-throughput functional genomics screen using the FLeXSelect human FL cDNA library identified orphan G protein-coupled receptor 3 (GPR3)^92^. GPR3 appears to promote complex assembly of γ-secretase, resulting in increased trafficking of the γ-secretase components and the mature γ-secretase complex to the cell surface and increased localization in lipid rafts, which eventually leads to an increase in Aβ generation^92^. Therefore, specific inhibition of γ-secretase in certain organelles or microdomains could be an attractive approach^91,93,94^, and a membrane-anchored version of a β-secretase transition state inhibitor reduced enzyme activity^95^.

### γ-Secretase structure

The catalytic residues of I-CliPs are located within transmembrane regions, and they hydrolyze the peptide bonds of their substrates in the transmembrane regions^96^. The I-CliP family can be categorized into aspartyl proteases (including γ-secretase and signal peptide peptidase), metalloproteases (site-2 protease, Eep), and serine proteases (Rhomboid, AarA)^97^. It had been challenging for γ-secretase structure studies due to its many subunits and transmembrane domains.

An electron microscopy study on the 3D structure of γ-secretase revealed that there is a low-density interior chamber and two pores (apical and basal pores), which allow for water molecule entry into the structure^98^. These pores for water molecules could explain this unusual intramembrane cleavage (peptide bond hydrolysis) by γ-secretase^98^. Aβ and AICD could be released through two pores into outer spaces (extracellular and cytosolic spaces, respectively)^98^. In 2015, single-particle cryoelectron microscopy (cryo-EM) revealed an atomic structure of γ-secretase in a substrate-free state with a 3.4 Å resolution^99^. In recent years, cryo-EM structures of the γ-secretase complex either bound to APP (C83) at a 2.6 Å resolution or Notch (Notch-100) at a 2.7 Å resolution have shown that PS1 undergoes conformational changes upon substrate binding^100–102^. Substrate-bound γ-secretase showed that the β-strand from the C-terminal of APP together with two APP-induced β-strands of PS1 form a hybrid β sheet, which guides γ-secretase cleavage for substrates^101^.

Nicastrin acts as a gatekeeper for the entry of γ-secretase substrates to block substrates with long extracellular domains^103^. APP can enter, either in whole or in part, at the substrate docking site between PS-NTF and PS-CTF to access the internal active site^104^. In other words, after a substrate binds to the docking site on PS, the substrate is moved into the S1′, S2′, and S3′ sites (three substrate binding pockets) in the active site of PS by lateral gating, and long Aβ peptides are produced^105^. Then, long Aβ peptides were cleaved by tripeptide trimming (Aβ49→46→43→40→37 or Aβ48→45→42→38) to release Aβ peptides (Fig. 2)^17^.

### γ-Secretase substrates

γ-Secretase has more than 140 substrates and substrate candidates in addition to APP, which are type 1 transmembrane proteins^106^. These substrates include APLP1 and APLP2 (together with APP, regulating synaptic plasticity and neuronal excitability), proteins involved in cell adhesion (N-cadherin, E-cadherin, CD44), the CSF1 receptor (protein tyrosine kinase), deleted in colorectal cancer (DCC, Netrin-1 receptor), ErbB4 (growth factor-dependent receptor tyrosine kinase), low-density lipoprotein receptor-related protein (endocytic receptor), Nectin-1α (adherens junction formation), Notch 1–4 (signaling receptors), Delta and Jagged (Notch ligands), p75 (neurotrophin co-receptor), and syndecan-3 (cell-surface proteoglycan co-receptor)^97,107^. γ-Secretase cleavage does not depend on the specific sequence of the substrate. Rather, it depends on ectodomain shedding^108^. In many cases, the intracellular domains (ICDs) released upon γ-secretase cleavage are involved in the regulation of gene transcription^97^.

**Fig. 4:**
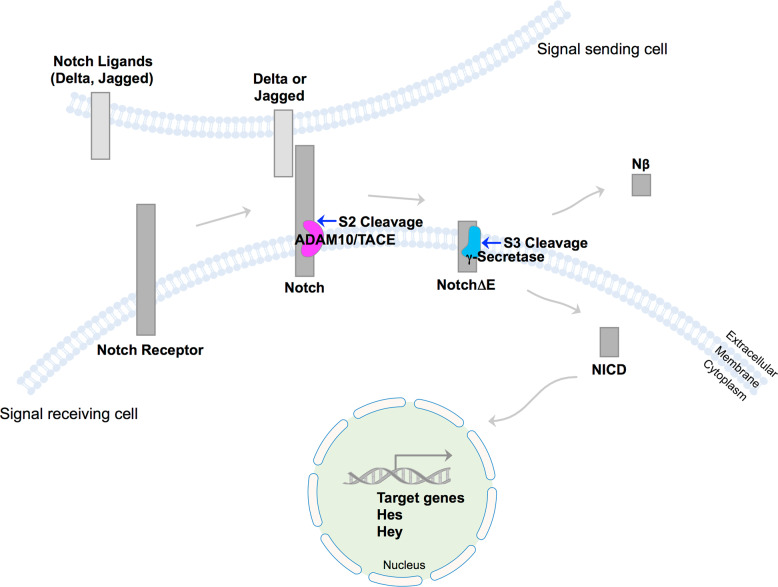
Notch processing. Notch ligands (ex. Delta, Jagged) from signal sending cells bind to Notch receptors (Notch 1–4) at signal receiving cells. Notch undergoes ectodomain shedding by ADAM metalloproteases (ex. ADAM10, TACE) at the extracellular S2 site (S2 cleavage). A membrane-bound truncated form of Notch, NotchΔE substrate, is further cleaved by γ-secretase at the S3 site (S3 cleavage) and releases Nβ and the Notch intracellular domain (NICD). NICD is translocated to the nucleus to regulate transcription genes such as Hes and Hey.

A well-known γ-secretase substrate, Notch, undergoes ectodomain shedding by metalloprotease at the S2 site, which is further cleaved by γ-secretase at the S3 site and releases the Notch intracellular domain (NICD) (Fig. 4)^97^. Rare genetic variants of *TREM2* (ex. R47H) are associated with AD^109^. The microglial surface receptor TREM2 and its adaptor protein DAP12 (TYROBP) cascade TREM2 signaling, which promotes phagocytosis^109^. After TREM2 undergoes ectodomain shedding by ADAM10, it was reported that TREM2-CTF can be cleaved by γ-secretase in cells^110^. The processing of several substrates by γ-secretase was investigated by ICD formation and the accumulation of substrate-C-terminal fragments (CTFs) by western blotting^111^. Ideally, an in vitro assay can confirm substrate cleavages^106^. Structurally, the β-strand region of several substrates (CD43, CD44, N-cadherin, ErbB4, and CD33) was aligned with the β-strand sequences of APP and Notch^112^. CD43 and CD44 closely resemble Notch 1, whereas N-cadherin, ErbB4, and CD33 share similar features with APP (C99)^112^. There is still much to be investigated for these substrate cleavages by γ-secretase and their physiological functions.

### Gain or loss of function of PS

The common feature of *PSEN1* or *PSEN2* FAD mutations is the increased Aβ42/40 ratio. However, it has been debated whether it is due to a gain or loss of PS function that results in an increased Aβ42/40 ratio^113^. An Aβ42/40 ratio increase could be due to increased Aβ42 production, decreased Aβ40 production, or a combination of both^111^. Analysis of the formation of substrate CTFs, ICDs, and Aβ species as the effect of FAD mutations of *PSEN1* or *PSEN2* on the cleavage of various γ-secretase substrates, such as APP, Notch, syndecan-3, N-cadherin, and β1-integrin, showed that different mutations had a varying effect on substrate processing, indicating “variable” or “partial” loss of PS protein function, and PS2 was less efficient than PS1^111^. Reconstitution of the PS protein from 138 PS1 FAD mutations with Aph-1aL containing γ-secretase mostly decreased the production of Aβ42 and Aβ40, increased the Aβ42/40 ratio, and suggested the loss of PS1 function^114^. However, these 138 PS1 FAD mutations also showed that different mutations displayed variations in Aβ42 or Aβ40 production (increase or decrease)^114^. In addition, further studies addressing the effect of PS FAD mutations on the structure of γ-secretase and how those conformational changes could affect the cleavage of different substrates by γ-secretase remain to be investigated. For instance, E280 in PS1 forms hydrogen bonds with Y159 and Y154^102^. PS1 E280A (the Columbian mutation) disrupts hydrogen bonds and causes a local conformational change^102^.

### Small molecules targeting γ-secretase

Over the years, small molecule inhibitors and modulators targeting γ-secretase have been developed as potential disease-modifying agents in AD. The main goal is to target γ-secretase and reduce toxic Aβ42 species while sparing other substrate cleavage processing by γ-secretase.

### γ-Secretase inhibitors

GSIs bind to the active site of PS and inhibit γ-secretase cleavage, thereby reducing total Aβ production. GSIs such as L-685,458^35,115^, BrA-1-Bt^34^, III-31C^116^, DAPT^117^, and Merck C^57^ as well as GSI-based chemical probes have been widely used to study γ-secretase. A GSI-based photoaffinity probe showed that <14% of PS1 is incorporated into active γ-secretase complexes and catalytically active while leaving the rest of PS1 in inactive γ-secretase complexes^118^. Thus, GSI-based chemical probes are critical to differentiating enzymatically active γ-secretase complexes from inactive complexes^119^. On the other hand, a co-immunoprecipitation study against γ-secretase complex components pulled down both active and inactive γ-secretase complexes.

In animal studies, GSIs successfully reduced Aβ production. DAPT decreased Aβ levels in the plasma, CSF, or brain of AD transgenic mice^117,120^. Chronic treatment with LY-411,575 in AD transgenic mice reduced Aβ but also inhibited Notch signaling, leading to side effects^121^. Semagacestat (LY-450,139) and avagacestat (BMS-708,163) in Tg2576 mice reduced Aβ production while increasing APP-CTF^122^. However, those GSIs impaired normal cognition in wild-type mice^122^. Begacestat (GSI-953) reduced Aβ levels in Tg2576 mice^123^.

In clinical trials, GSIs such as semagacestat (LY-450,139, Eli Lilly) and avagacestat (BMS-708,163, Bristol-Myers Squibb) reduced Aβ production in AD patients^124,125^. However, the multitude of γ-secretase substrates has made the development of clinically useful inhibitors difficult. Due to the decreased Notch signaling and the accumulation of APP-CTFs^122^, side effects such as the risk of skin cancer and infection, gastrointestinal bleeding, and worsening cognition led to the pause of clinical trials^5,124,125^. Therefore, these GSIs are nonselective and inhibit both APP and Notch^121,124,126^. Avagacestat was reported as a “Notch-sparing” GSI and was shown to have a higher selectivity for APP over Notch cleavage^127^. However, avagacestat was suggested to be nonselective later based on poor Notch-sparing activity^122,128^ and its binding site as PS1-NTF^128^. Another “Notch-sparing” GSI, begacestat (GSI-953, Wyeth/Pfizer), was also discontinued in phase I clinical trial, and the reasons are unclear^129^. Another concern regarding GSI treatment is the Aβ rebound effect. GSIs at lower doses increased Aβ levels, and discontinuation of GSI treatment was observed with a rebound of Aβ levels^130,131^. These GSIs target PS1-NTF^128^. GSIs have been repurposed in the cancer field for Notch signaling inhibition and are currently in clinical trials.

### γ-Secretase modulators

Instead of inhibiting the whole γ-secretase activity, modulating γ-secretase activity by γ-secretase modulators (GSMs) has been tested. GSMs are more attractive disease-modifying agents than GSIs because GSMs (1) inhibit selectively aggregation-prone Aβ42 production, (2) increase shorter Aβ37 or Aβ38 species, (3) do not affect the total Aβ production and the accumulation of APP-CTF, and (4) spare Notch processing^132^.

Nonsteroidal anti-inflammatory drugs (NSAIDs), such as ibuprofen, indomethacin, and sulindac sulfide, were found to modulate γ-secretase and represent first-generation GSMs (NSAID-derived carboxylic acid GSMs)^132^. These NSAIDs lowered Aβ42 and increased Aβ38 without affecting Notch cleavage^133^. This Aβ modulation was not due to the inhibition of cyclooxygenase activity, the pharmacological target of NSAIDs^133^. Sulindac sulfide treatment showed a varying degree of Aβ42 reduction levels while increasing high Aβ38 levels in cells overexpressing PS1 FAD mutants^134^.

Second-generation GSMs were developed to improve in vivo potency and blood-brain penetrance, including NSAID-derived carboxylic acid GSMs, non-NSAID-derived imidazole GSMs, and natural product-derived GSMs^132^. Acid GSMs decrease Aβ42, increase Aβ38, and have little effect on Aβ40 levels, total Aβ levels, and NICD production^132^. GSM-1 (acid GSM, GSM-2, and GSM-10 h as close analogs) reduced Aβ42 in many PS mutants but did not reduce Aβ42 levels in cells overexpressing PS1 L166P or PS2 N141I mutants^134,135^. GSM-2 improved memory in Tg2576 mice and did not affect cognition in wild-type mice^122^. Acute and subchronic administration of GSM-10 h decreased Aβ42 with no effect on Notch signaling, and there was no Aβ rebound effect and no accumulation of APP-CTFs (C83 and C99)^136,137^. E2012 (imidazole GSM) decreased Aβ42, Aβ40, and Aβ39 and increased Aβ37 and slightly Aβ38 without affecting Notch processing^138^.

Several GSMs have entered AD clinical trials. Tarenflurbil ((R)-flurbiprofen, NSAID GSM, Myriad Genetics & Laboratories) failed in phase III clinical trial due to lack of efficacy^139^. However, there were no Notch inhibition-related adverse effects^140^. A safety study in rats indicated that E2012 (Eisai) induced cataracts by inhibiting the final step in cholesterol biosynthesis^141^. Therefore, E2012 was withdrawn from the phase I clinical trial, and Eisai pursued E2212^119^. E2212 has a better safety profile than E2012 and was evaluated for safety, tolerability, pharmacokinetics, and pharmacodynamics in healthy subjects in phase I clinical trial^140^. The most common adverse effect was diarrhea^140^. PF-06648671 (Pfizer) was well tolerated at single doses in healthy subjects, lowered plasma Aβ40 and Aβ42, and increased Aβ37 and Aβ38^142^. However, this small molecule was discontinued due to Pfizer’s discontinuation of R&D in neurology in 2018. EVP-0962 (NSAID GSM, Forum Pharmaceuticals, Inc.) was discontinued after the phase II clinical trial, and the results were not reported^129^. CHF5074 (acid GSM, CereSpir Incorporated, Chiesi Pharmaceuticals, Inc.) was first thought of as a GSM and improved memory and reduced microglial activation in Tg2576 mice^143^. CHF5074 lowered soluble CD40 ligand levels (microglia activation marker)^144^ and is considered a microglia modulator^145^. The natural product GSM NIC5-15 (Humanetics Pharmaceuticals Corporation), which is found in soy, plants, and fruits, reduced Aβ production without affecting Notch processing (ALZFORUM, http://www.alzforum.org)^129^. Amyloid PET in APP-Swe transgenic mice revealed that chronic treatment with RO5506284 reduced de novo amyloid plaque formation^146^. The GSM-based brain imaging agent [^11^C]SGSM-15606 was also developed and showed γ-secretase imaging in the brains of mice and macaques^147^. Recently, chronic treatment with GSM UCSD-776890 in PSAPP mice reduced amyloid deposition and microgliosis^148^.

### GSI and GSM-binding sites

GSIs bind to the active site of PS and inhibit the γ-secretase cleavage of APP and Notch. The mechanism of how GSMs shift Aβ cleavages from longer Aβ peptides to shorter Aβ peptides and where GSMs bind in γ-secretase complexes are still poorly understood^129^. Moreover, the physiological role of shorter Aβ peptides (Aβ38 or Aβ37) is not well understood. To understand the mechanism of action of GSMs in modifying γ-secretase activity, researchers have investigated GSM target proteins. Studies have shown that NSAID GSMs bind to APP or γ-secretase^132^. GSM-1-based photoaffinity probes label PS1 or PS1-NTF, and E2012 targets PS1-NTF in γ-secretase complexes^149–151^. Further identification of potential GSM-binding proteins is important to understand the modulation of γ-secretase and to avoid possible side effects of GSMs in clinical trials.

**Fig. 5:**
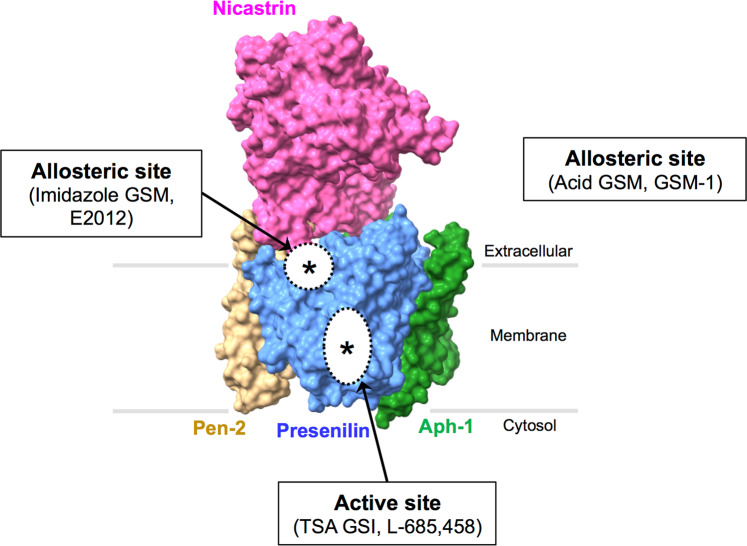
GSI and GSM-binding sites on PS. Based on cryo-EM structure studies by Yang et al., there are different binding sites for the active binding site for the transition state analog GSI (TSA GSI) (ex. L-685,458) and for the allosteric binding site for imidazole GSM (ex. E2012) (indicated by asterisks). Based on biochemical studies, there might be an additional allosteric binding site for acid GSM (ex. GSM-1) in PS. Note that the structure of acid GSM-bound γ-secretase has not yet been resolved by cryo-EM. Presenilin (blue), nicastrin (magenta), Aph-1 (green), and Pen-2 (yellow). Rendered from Protein Data Bank entry 7D8X. Structural figures were prepared with UCSF ChimeraX 1.2.5.

Various chemical probes have been developed to identify the multiple binding sites within the active γ-secretase complex. The “photophore walking” approach to modifying GSI-based photoaffinity probes with a benzophenone group on P2, P1, P1′, and P3′ of L-685,458 (GSI compounds: L646, GY4, JC8, and L505, respectively) can label subsites such as S2, S1, S1′, and S3′ in the active site of the γ-secretase complex^149,152^. GSI GY4 photolabeling in the presence of GSM-1 altered the S1 subsite of PS1 and increased GY4 labeling^149^. These results indicated that GSM-1 (acid GSM) caused an active site shape change (conformational change) in the γ-secretase complex and that there is a GSM-1 allosteric binding site on PS1 apart from the GSI L-685,458 binding site (Fig. 5)^149^. Photolabeling by E2012-Bpyne (imidazole GSM E2012-based photoaffinity probe) also revealed PS1-NTF as the imidazole GSM-binding site within γ-secretase complexes (Fig. 5)^150^.

In summary, biochemical studies suggest that distinct GSI and GSM modulation sites exist in PS of the γ-secretase complex: binding sites for TSA GSI and allosteric GSMs (acid GSM and imidazole GSM, respectively) (Fig. 5)^132^. These different classes of small molecules occupy different distinctive sites within the γ-secretase complex. Therefore, they interact and induce conformational changes in γ-secretase complexes, which lead to different Aβ cleavages^132^. For example, E2012-BPyne shows enhanced labeling of PS1-NTF in the presence of GSI L-685,458^150^. Recently, cryo-EM structure studies confirmed the different binding sites for TSA GSI (L-685,458) and imidazole GSM (E2012) found in the γ-secretase complex (Fig. 5)^112^. L-685,458 binds the active site of PS1, while the imidazole GSM E2012 binds to the allosteric binding site in PS1^112^. For a non-TSA GSI, semagacestat occupies the same location as APP (C99) and Notch (N100)^112^. It suggests that semagacestat could block hybrid β sheet formation between substrates and PS1, therefore inhibiting substrate cleavages^112^. Another non-TSA GSI (avagacestat) also occupies a similar binding site as semagacestat except with some variations^112^. L-685,458, semagacestat, and avagacestat share the same binding pocket in PS1, whereas L-685,458 has an additional unique binding pocket^112^. Co-incubation with L-685,458 and E2012 also showed that E2012 binds to the interface between Nct and PS1 (Fig. 5)^112^. Yang et al.^112^ suggested that GSIs and GSMs could be used in combination for a synergistic effect, and this structural information could also improve the design of substrate-selective small molecules for AD.

### γ-Secretase modulatory proteins (GSMPs)

Bateman et al.^153^ reported that Aβ production and clearance rates per hour in human CSF are 7.6% and 8.3%, respectively. Sporadic AD (SAD) human brain gray matter has 4.8 mg more total Aβ than healthy controls^154^. This gives the estimated Aβ accumulation rate in the brain ~28 ng/hour, and a 2–5% slight increase in Aβ deposition could lead to AD in ~20 years^154^. For FAD, PS FAD mutations increase the ratio of Aβ42/40, which leads to AD over several decades^155^. Therefore, reducing Aβ production by a few percent^154^ by modulating γ-secretase activity with GSMs and/or other means could be therapeutic for AD patients.

Although PS, nicastrin, Aph-1, and Pen-2 are essential subunits for γ-secretase activity^50^, it is plausible that other transiently binding proteins could regulate γ-secretase activity and/or substrate specificity in different types of tissues, cells, or subcellular organelles. In addition, the reported size of the γ-secretase complex varies between 200 and 2000 kDa^50,52,55^_,_ indicating that there is a possibility of unknown components being present in the γ-secretase complex. Moreover, most of the γ-secretase complexes are inactive, while <14% are enzymatically active^118^. How the activation of inactive γ-secretase complexes occurs is still unclear^119^. It is possible that the binding of GSMPs could shift γ-secretase complexes from inactive to active. Thus, these GSMPs could be new targets to modulate γ-secretase activity in AD. Over the years, several GSMPs have been identified by using different methods: multiple purification columns followed by amino acid sequencing^156^, IP followed by mass spectrometry (MS)^62^, and tandem affinity purification^157^. To capture GSMPs in enzymatically active γ-secretase complexes, researchers used GSI pulldown or GSM photolabeling followed by MS^158,159^. The following are some of the GSMPs reported.

### CD147

CD147 (also known as basigin, extracellular matrix metalloproteinase inducer (EMMPRIN)) is a transmembrane glycoprotein with two Ig-like domains, and CD147 was initially identified as a regulatory subunit of γ-secretase^156^. CD147 is ubiquitously expressed in various cells and tissues^160^ and is suggested to be involved in many biological functions, such as neural-glial cell interactions, reproduction, neural function, inflammation, protein trafficking, and tumor invasion^156^. The deletion of CD147 in mice has resulted in severe defects in nervous system development, spatial learning deficits, and working memory deficits^161^. Co-immunoprecipitation with anti-PS1-CTF and nicastrin antibodies indicated that CD147 is present in the γ-secretase complex^156^. Suppression of CD147 expression by siRNA resulted in dosage-dependent increases in the Aβ40 and Aβ42 levels without changes in the expression levels of the other γ-secretase components or APP substrates^156^. However, it was later questioned whether CD147 is associated with the γ-secretase complex. GSI pulldown using Merck C (biotinylated GSI L-685,458) did not co-purify CD147, indicating that CD147 is not engaged in active γ-secretase complexes^162^. Vetrivel et al.^163^ also reported that CD147 degrades Aβ and that it is independent of γ-secretase activity.

### TMP21

TMP21 (also known as p23) was identified by immunoprecipitation with an anti-PS antibody followed by MS^62^. TMP21 is a type I transmembrane protein^164^, is a member of the p24 cargo-protein family^164^ and is involved in protein transport and quality control in the ER and Golgi^165^. When TMP21 was suppressed by siRNA, Aβ production (Aβ40 and Aβ42) was increased^62,166^. TMP21 might have two pools. The major pool joins the p23 cargo family without affecting Aβ production, and the minor makes a complex with PS1^62^. Since TMP21 siRNA affects Aβ production (γ-cleavage site) but not AICD or NICD production (ε-cleavage site), TMP21 in the minor pool regulates only γ-cleavage^62^. The transmembrane domain of TMP21 interacts with γ-secretase to alter γ-cleavage^167^. Later, it was questioned whether TMP21 is truly a GSMP. It was reported that TMP21 also plays a role in APP trafficking, which affects Aβ production^168^. In addition, GSI Merck C (L-685,458 derivative) using detergent-solubilized human cell line membranes did not pulldown TMP21, suggesting that TMP21 might not be a part of active γ-secretase complexes^162,168^. However, TMP21 was found to be associated with endogenous active γ-secretase complexes using GSI GCB (L-685,458 derivative) in the brain^159^ and brain lipid rafts^169^. This discrepancy between cell lines and brain studies might be because TMP21 in the minor pool is transiently associated with γ-secretase, and different materials and methods were used^62,159,162,169^. Interestingly, it was easier to detect TMP21 in lipid raft-associated γ-secretase than in detergent-solubilized membranes from the brain^159,169^. For AD, TMP21 protein expression levels (normalized to nicastrin protein levels) were decreased in the brains of SAD and FAD patients^170^, and the *TMP21* SNP rs12435391 was associated with SAD^171^.

### GSMPs in membrane microdomains

Previously, it was reported that γ-secretase is localized at membrane microdomains called lipid rafts^86,89,90,172^. Several GSMPs in lipid raft-like microdomains or lipid rafts were identified. Proteins in the tetraspanin web were identified as GSMPs: EWI-F, CD81, CD98hc, and CD9^157^. Members of this family form lipid raft-like microdomains in cellular membranes^157^ and have a role in intracellular and intercellular processes, cell fusion, cell proliferation, adhesion, and migration^173^. EWI proteins (EWI-F) form a primary interaction with tetraspanins (CD81), followed by a secondary interaction with integrins (β1-integrin), and the tetraspanin web makes a tertiary interaction with the γ-secretase complex^157^. Suppressing *CD81*, *EWI-F,* or *CD98hc* by siRNA decreased Aβ production^157^. CD81, CD9, and EWI-F are in γ-secretase complexes, as shown by Aβ production in co-immunoprecipitates^157^. The α-secretase ADAM10 was also associated with tetraspanins for the cleavage of TNF-α and epidermal growth factor (EGF)^174^_,_ and a new APP processing model by α- and γ-secretases in the tetraspanin web was proposed^175,176^.

GSI pulldown using lipid rafts from the brain identified several endogenous GSMPs that regulate active γ-secretase. Voltage-dependent anion channel 1 (VDAC1, also known as porin) and contactin-associated protein 1 (CNTNAP1, also known as Caspr) are associated with active γ-secretase complexes in brain lipid rafts, and silencing those genes in HEK293-APP695 cells decreased Aβ production^169^. VDAC is a major protein at the outer mitochondrial membrane^177^. A new member of the VDAC family, B-36 VDAC at 36 kDa, was found during the purification of the GABA_A_ receptor and was localized at the membrane of nonpyramidal neurons in the human prefrontal cortex^178^. VDAC at the plasma membrane of neurons was also reported^179^. VDAC accumulates around amyloid plaques in APP/PS1 Tg mice^177^. Nitrated VDAC1 protein was increased in the hippocampus of AD brains^180^, and VDAC and estrogen receptor alpha in caveolae are highly expressed in AD human brains^179^. CNTNAP1 and contactin are essential for forming the paranodal junction in myelinated axons^181^. Contactin interacts with APP in neurons and brains^182,183^. CNTNAP1 interacted with APP, and overexpression of CNTNAP1 decreased Aβ production in HEK293 cells overexpressing APP V717F (Indiana mutant)^184^. Erlin-2, which is known to be located at lipid rafts from the ER, is also engaged in active γ-secretase from lipid rafts to regulate Aβ levels^185^. VDAC1, Erlin-1, and Erlin-2 were previously shown to interact with PS^157^. Flotillin-2, syntaxin-binding protein 1, solute carrier family 2 member 3, and growth-associated protein 43 were also found as γ-secretase-associated proteins in lipid rafts^186^.

### Endogenous GSMPs in the brain

Using a biotinylated GSI, GCB (GSI coupled to biotin via a cleavable linker, L-685,458 derivative), in the brain instead of using overexpressed cell lines identified several endogenous GSMPs. Previously, a yeast two-hybrid screening identified a synaptic plasma membrane protein, syntaxin 1A, which binds to PS1^187^. Less than 1% of syntaxin 1 associates with endogenous active γ-secretase complexes in brain membranes^159^ and brain lipid rafts^169^. Proton myoinositol cotransporter (SLC2A13) is another endogenous brain GSMP that regulates Aβ production without affecting Notch processing^188^.

GSI GCB also pulled down several endogenous GSMPs from synapses in the brain, such as NADH dehydrogenase iron-sulfur protein 7 (NDUFS7) from synaptic vesicles and tubulin polymerization promoting protein (TPPP) from synaptic membranes^189^. Silencing NDUFS7 decreased Aβ levels, while TPPP increased Aβ production, and both proteins were co-immunoprecipitated with Nct and PS1-CTF in the human brain^189^. Previously, it was shown that monoamine oxidase B (MAO-B) is increased in AD brains and platelets^190^. Overexpression of MAO-B increased Aβ production, and MAO-B was associated with active γ-secretase^191^. MAO-B levels were increased in neurons of AD human brains^191^.

### γ-Secretase activating protein

The treatment for chronic myeloid leukemia, Gleevec (an anticancer drug, imatinib mesylate, STI571), was shown to reduce Aβ production but spare Notch cleavage^192^. A biotinylated derivative of imatinib identified GSAP (γ-secretase activating proteins)-16 kDa, and GSAP is the C-terminal region of an uncharacterized protein, pigeon homologue protein (PION)^63^. GSAP-16 kDa, γ-secretase, and APP-CTF form a tertiary complex^63^. Knockdown (KD) of GSAP by siRNA reduced Aβ production and did not change NICD production, and recombinant GSAP-16 kDa increased Aβ production^63^. KD of GSAP by crossing AD X 2 mice with doxycycline-inducible GSAP RNAi mice resulted in the reduction of Aβ and amyloid plaques in the brain^63^. Knockout (KO) of GSAP also decreased Aβ production while sparing Notch cleavage^193^. Overexpression of FL GSAP in GSAP KO cells increased Aβ generation^193^. Treatment with imatinib in 3XTg mice (mutant APP, mutant PS1, and mutant MAPT) decreased GSAP-16 kDa protein, Aβ production, brain Aβ deposits, and phosphorylated tau^194^.

However, the relationship between GSAP and γ-secretase for Aβ generation was later questioned by several groups^195^. Hussain et al.^195^ reported that KD of GSAP decreased Aβ levels, but overexpression of GSAP-16 kDa did not increase Aβ production, and APP-CTF/PS1-CTF complexes were immunoprecipitated without GSAP. Hussain et al.^195^ suggested that Aβ reduction by KD of GSAP might be due to some effects on the trafficking or assembly of γ-secretase but not a direct effect of GSAP on γ-secretase. In addition, imatinib did not decrease Aβ generation in cell lines and in vivo while sparing Notch processing in cell lines. Another study also showed that, unlike GSI L-685,458, imatinib treatment did not inhibit Aβ production in cell lines, mouse primary neurons, and differentiated human embryonic stem cells^196^. In humans, imatinib treatment in chronic myeloid leukemia patients for up to 12 months also did not result in an Aβ decrease in plasma^196^. In contrast, overexpression of GSAP-FL in GSAP KO cells rescued γ-secretase activity, and the dual GSI photoprobe L631 for PS1-NTF and PS1-CTF labeled PS1-NTF, PS1-CTF, and FL PS1 when GSAP-FL was overexpressed in GSAP KO cells compared to KO cells^193^. This result suggested that the presence of GSAP aligned PS1-NTF and PS1-CTF in a specific confirmation with higher γ-secretase activity for Aβ cleavage^193^. In human brains, an immunohistochemistry study showed that GSAP-positive deposits are present both in control and AD brains, while the quantification of GSAP-positive deposits is higher in AD brains, and these GSAP-positive deposits are closely localized to PS1 and Aβ deposits in AD brains^197^. It was also reported that the *GSAP* SNP rs4727380 was associated with APOE4 noncarriers of AD patients from Han Chinese in a small sample size^198^.

### GSMPs induced by other factors

Hif-1α was identified as a GSMP for Notch processing. Hif-1 expression is upregulated by aging in the frontal cortex of the human brain^199^, and stroke increases the risk for dementia^200^. Brain ischemia/hypoxia-induced Aβ deposits in the human brain^201^. The BACE1 gene contains a hypoxia response element (HRE) in the promoter region, and hypoxia increases BACE1 protein expression as well as β-secretase cleavage for APP^202^. A transcription factor, Hif-1α (hypoxia-inducible factor-1α), works as an oxygen sensor, and Hif-1α is degraded by the ubiquitin-proteasome system under normoxia^203^. Under hypoxia, the canonical hypoxic response leads to the binding of Hif-1α/Hif-1β to HRE elements in the promoter regions of several genes, such as vascular endothelial growth factor, erythropoietin (Epo), and glucose transporters 1, for angiogenesis, erythropoiesis, and energy metabolism^203^. Gustafsson et al.^204^ discovered crosstalk between the noncanonical pathway of Hif-1α and Notch signaling. Under hypoxia, Hif-1α binds to the NICD and induces Notch downstream genes such as Hes and Hey for the undifferentiated cell state in the stem cell population^204^. Villa et al.^64^ found that hypoxia also increases active γ-secretase complex formation and upregulates γ-secretase activity to cleave Notch. Nontranscriptional Hif-1α converts the pool of inactive γ-secretase to active γ-secretase, and GSI-34 decreases hypoxia-induced cell invasion and metastatic progression in cells and animal models of breast cancer^64^.

Another environmental factor, such as stress, activates G protein-coupled receptors (GPCRs), such as β2-adrenergic receptor (β2-AR) and δ-opioid receptor^205^. β2-AR agonists stimulate Aβ production via (1) the association with PS1, (2) the endocytosis of the receptor, and (3) the trafficking of γ-secretase to late endosomes and lysosomes^205^. High-throughput functional genomics screening identified another GPCR, orphan GPR3, that modulates Aβ production^92^. Overexpression of GPR3 increased (1) the expression of mature γ-secretase complexes at 440 kDa, (2) the localization of γ-secretase complexes to lipid rafts, and (3) Aβ and AICD production, but (4) did not change Notch cleavage^92^. Crossing APP/PS1 mice with GPR3 KO mice also decreased Aβ production, and GPR3 was expressed in the brains of SAD patients^92^.

GPCRs require adaptor proteins such as arrestins to prevent further G protein-mediated signaling^206^. β-Arrestin1 is highly expressed in the brain^206^, and β-arrestin1 KO mice exhibit reduced Aβ production and spared Notch cleavage^207^. β-arrestin1 interacts only with Aph-1 in γ-secretase, and overexpression of β-arrestin1 enhanced mature γ-secretase complex formation at 440 kDa^207^. KO of β-arrestin1 in APP/PS1 mice decreased Aβ production and improved memory deficits^207^. Stress-associated endoplasmic reticulum protein 1 (SERP1) was also reported to regulate the assembly of γ-secretase complexes and contribute to Aβ pathogenesis^208^. SERP1 interacts with the Aph-1a/Nct subcomplex of γ-secretase and increases γ-secretase activity for Aβ generation but reduces Notch processing^208^.

### GSMP in neuroinflammation

**Fig. 6:**
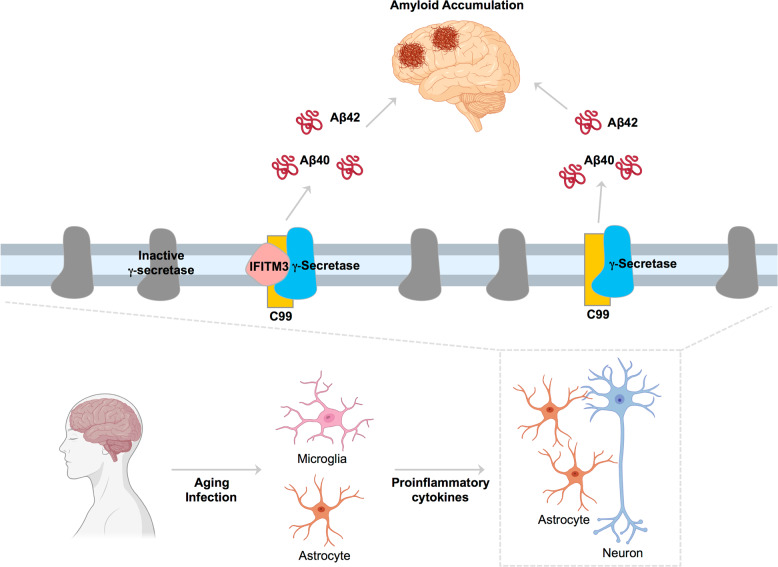
Aβ production by IFITM3-γ-secretase complexes. Normally, active γ-secretase cleaves its substrate to release Aβ. Under inflammatory conditions such as aging and infection, proinflammatory cytokines are induced by microglia and astrocytes. These cytokines upregulate IFITM3 protein expression in astrocytes and neurons, which in turn increases the processing of APP-CTF (C99) by active IFITM3-γ-secretase complexes to produce Aβ40 and Aβ42. The accumulation of amyloid leads to amyloid build-ups in the brain. Note that less than 14% of γ-secretase complexes are enzymatically active, while the rest are inactive.

Recently, IFITM3 (interferon-induced transmembrane protein 3, also known as fragilis) was identified as an imidazole GSM, E2012, binding protein^158^. Photolabeling with E2012-BPyne (an E2012-based photoaffinity probe) followed by LC-MS/MS identified IFITM3 at 15 kDa as a GSMP^158^. IFITM3 plays a role in innate immunity as an antiviral protein that restricts viral protein entry into host cell membranes by inhibiting membrane fusion^209^. IFITM3 KO mice are susceptible to viral infections^210^. Previously, microarray analysis and RT-PCR showed 19.9- and 3.4-fold increases in *IFITM3* in SAD brains^211^. Hur et al.^158^ showed that IFITM3 binds to PS1-NTF in active γ-secretase complexes and regulates γ-secretase activity for Aβ production (Aβ40 and Aβ42) (Fig. 6). KD or KO of IFITM3 decreased Aβ production, and overexpression of IFITM3 increased Aβ levels in IFITM3 KO cells^158^. Moreover, crossing IFITM3 KO mice with 5XFAD Tg mice decreased Aβ production and amyloid plaque formation in the cortex and hippocampus^158^. Aging mouse models also showed increased IFITM3 levels, γ-secretase activity, and active IFITM3-γ-secretase complex formation levels by aging^158^. A positive correlation between the amount of active IFITM3-γ-secretase complexes and the high γ-secretase activity resulting in high Aβ production was shown in the subsets of SAD patient brains expressing high IFITM3 protein levels^158^. Proinflammatory cytokines such as Type I IFN or Type II IFN can induce IFITM3 protein expression, increase the engagement of IFITM3 in active γ-secretase complexes, and increase Aβ production in mouse primary cortical neurons^158^. This result shows the direct link between inflammation and Aβ production via IFITM3-γ-secretase in neurons^158,212,213^. IFITM3 modulates γ-secretase under inflammation in neurons and astrocytes and may contribute to aging and the pathogenesis of AD^158^. The “antimicrobial protection hypothesis of AD” proposes that Aβ is beneficial as an antimicrobial peptide and that Aβ fibrilization entraps bacteria and viruses as an innate immune response to pathogens^214^. The involvement of IFITM3 in Aβ production might suggest the role of the “neuronal innate immune response” against pathogens, and Aβ has resulted as a protective pathway against infection^212^. At the same time, the accumulation of Aβ poses a risk of developing AD^158^. Further studies on regulating other γ-secretase substrates by IFITM3-γ-secretase complexes are needed to understand possible adverse effects when targeting IFITM3 in AD.

## Conclusion

Aβ is the key driver in AD according to the amyloid cascade hypothesis. Since γ-secretase cleaves its immediate substrate APP-CTF to release Aβ, which causes AD, and its unique biology as a transmembrane protein complex enzyme is still much to be learned, γ-secretase is still interesting to study. How γ-secretase cleaves over 100 substrates and how those signaling cascades could result in different physiological functions remain to be determined in the future. To validate γ-secretase as an Aβ modifying drug, further studies on the regulation/modulation of γ-secretase by GSMs and transiently binding GSMPs are needed. In addition, the effects of GSMs and GSMPs on different substrate processing need to be elucidated. This knowledge could advance the development of AD-modifying drugs by selectively inhibiting APP processing by γ-secretase.
